# Lessons learned after 20 years' experience with penile fracture

**DOI:** 10.1590/S1677-5538.IBJU.2019.0367

**Published:** 2020-02-20

**Authors:** Rodrigo Barros, Daniel Hampl, Andre Guilherme Cavalcanti, Luciano A. Favorito, Leandro Koifman

**Affiliations:** 1 Hospital Municipal de Souza Aguiar Serviço de Urologia Rio de JaneiroRJ Brasil Serviço de Urologia, Hospital Municipal de Souza Aguiar, Rio de Janeiro, RJ, Brasil;; 2 Universidade Federal do Estado do Rio de Janeiro - Unirio Disciplina de Urologia Rio de JaneiroRJ Brasil Disciplina de Urologia, Universidade Federal do Estado do Rio de Janeiro - Unirio, Rio de Janeiro, RJ, Brasil;; 3 Universidade Estadual do Rio de Janeiro - UERJ Unidade de Pesquisa Urogenital Rio de JaneiroRJ Brasil Unidade de Pesquisa Urogenital, Universidade Estadual do Rio de Janeiro - UERJ, Rio de Janeiro, RJ, Brasil

**Keywords:** Penile Erection, Penis, Learning

## Abstract

**Objective:**

To report our experience over the past 20 years in the diagnosis and surgical treatment of penile fracture (PF).

**Materials and methods:**

Between January 1997 and January 2017, patients with clinical diagnosis of PF were admitted to our facility and retrospectively assessed. Medical records were reviewed for clinical presentation, etiology and operative findings. Postoperative complications, sexual and urinary function were evaluated.

**Results:**

Sexual trauma was the main etiological factor, responsible for 255 cases (88.5%): 110 (43.1%) occurred with the “doggy style” position, 103 (40.3%) with “man on top” position, 31 (12.1%) with the “woman on top” position and 11 (4.3%) in other sexual positions. The most common findings in the clinical presentation were hematoma, in all cases and detumescence in 238 (82.6%). Unilateral corpus cavernosum injuries were found in 199 (69%) patients and bilateral in 89 (31%) patients. Urethral injuries were observed in 54 (18.7%) cases. Nine (14.7%) patients developed erectile dysfunction and eight (13.1%) had penile curvature. Only two (3.7%) patients had complications after urethral reconstruction.

**Conclusions:**

PF has typical clinical presentation and no need for additional tests in most cases. Hematoma and immediate penile detumescence are the most common clinical findings. Sexual activity was the most common cause. The ‘doggy style’ and ‘man-on-top’ was the most common positions and generally associated with more severe lesions. Concomitant urethral injury should be considered in cases of highenergy trauma. Surgical reconstruction produces satisfactory results, however, it can lead to complications, such as erectile dysfunction and penile curvature.

## INTRODUCTION

Penile fracture (PF) is a relatively uncommon form of urologic trauma. Vaginal intercourse is the most common cause of PF (1), but non-coital etiology (masturbation or penile manipulation) is also reported, especially in some Middle Eastern countries (2). Generally, patients report hearing a cracking noise during sexual activity, followed by immediate pain and penile detumescence, in addition to the emergence of large edema and hematoma, leading to an ‘eggplant deformity’ (3). Diagnosis is typically clinical. However, in doubtful cases, additional examinations such as ultrasonography (USG) and magnetic resonance imaging (MRI) can be used for diagnostic confirmation (4). The treatment is usually surgical, where closure of the tunica albuginea is used to prevent sequelae such as erectile dysfunction (ED), curvature and painful erections (5).

The aim of this study is to report our experience over the past 20 years in the diagnosis and surgical treatment of PF along with the long-term outcomes.

## MATERIAL AND METHODS

Between January 1997 and January 2017, 285 patients with clinical diagnosis of PF were admitted to our facility and retrospectively assessed. Our institution is the biggest urologic emergency unit in Rio de Janeiro, a metropolitan area in Brazil with more than 6 million inhabitants.

The medical records were systematically reviewed for epidemiological data, history and clinical presentation, etiology, and operative findings. Primary diagnosis assessment was performed through clinical history and physical examination. Complementary imaging methods such as USG and MRI of the penis were performed only in doubtful cases. Retrograde urethrography (RGU) was performed in selected cases when urethral injury was suspected.

All patients underwent surgical treatment immediately after diagnosis. The technique standardized in our institution, as previously described (6), is a circular sub-coronal incision and degloving of the penis, followed by debridement and synthesis of the injury, using simple interrupted sutures of 3-0 polyglactin. The urethral injuries are repaired using simple interrupted sutures of 5-0 polyglactin placed under a Foley catheter. Postectomy is performed routinely in all uncircumcised patients.

From the third month after surgery, all patients with urethral lesion answered the IPSS questionnaire (International Prostate Symptom Score) and underwent uroflowmetry. Patients having altered IPSS or uroflowmetry underwent RGU to exclude or confirm urethral stenosis. Six months after surgery, patients who reported having acquired curvature underwent a drug-induced erection test using alprostadil 10mcg, to evaluate the exact type and degree of curvature. The evaluation of the postoperative erectile function was performed by completing the International Index of Erectile Function (IIEF-5). Penile color duplex doppler ultrasound (CDDU) was performed for those who had persistent ED to obtain a precise etiological diagnosis.

Regarding statistical analyses, correlations between target events were assessed using Pearson's correlation coefficient. The chi-squared or Fisher's exact test, when appropriate, was employed for contingency table analyses. P-value <0.05 was considered significant.

The experimental protocol was approved by our institution's ethics and human research committee. The patients who refused to sign informed consent form or those who underwent incomplete follow-up were excluded.

## RESULTS

From a total of 285 patients evaluated in this study, we identified 288 cases of PF (3 patients presented an additional PF after the primary episode). The patient's age ranged from 18 to 69 years (mean 38.2 years). Time elapsed between trauma and hospital admission ranged from 2 to 504 hours (mean 18.5 hours).

Investigation of the mechanism of injury revealed sexual trauma as the main etiological factor, responsible for 255 cases (88.5%). Masturbation was reported by nine patients (3.1%). For non-sexual injury mechanisms, we found penile manipulation in 18 cases (6.2%) and rolling in bed in one case (0.3%). Five patients (1.7%) refused to provide data on the injury mechanism.

Of the 255 cases in which the etiology was sexual intercourse, 110 (43.1%) cases occurred with the “doggy style” position, 103 (40.3%) with “man on top” position, 31 (12.1%) with the “woman on top” position and 11 (4.3%) in other sexual positions.

The most common findings in the clinical presentation were hematoma in all cases (100%), detumescence in 238 (82.6%), a snapping sound in 220 (76.3%), pain in 191 (66.3%), urethral bleeding in 37 (12.8%), and acute urinary retention in one (0.3%). All patients with urethral bleeding or acute urinary retention had experienced some degree of urethral injury.

Imaging tests were performed on 46 (16.1%) patients, of whom 19 (6.6%) underwent USG and two (0.7%) underwent MRI of the penis. The remaining 25 (8.7%) patients with suspected urethral injury underwent retrograde urethrography and diagnostic confirmation was achieved in all cases.

**Figure 1:**
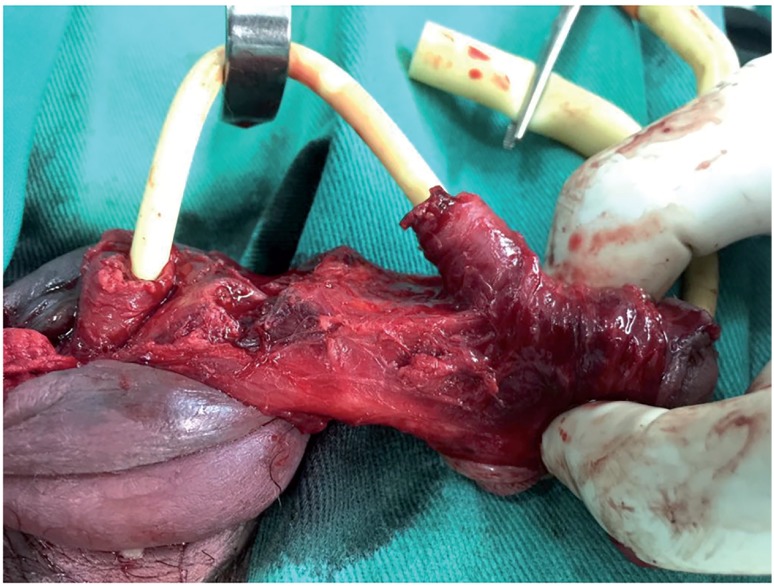
Complete rupture of the urethra associated with bilateral injury in the corpus cavernosum.

Unilateral injuries of the corpus cavernosum were found in 199 patients (69%) and bilateral injuries were identified in 89 (31%) patients. Urethral injuries were observed in 54 cases (18.7%), including 39 (13.5%) partial injuries and 15 (5.2%) total injuries. The complete rupture of the urethra was associated with bilateral injury in the corpus cavernosum in 100% of cases (Figure-1). Of three patients with refracture, all presented the second episode with injury at the same point as the primary repair and contralateral involvement was observed in only one case. Demographic data and intra-operative findings are summarized in Table-1.

**Table 1:** Demographic data and intra-operative findings.

Cases (N)		288
Average age (years)		38.2 (18-69)
**Etiology**		
	Sexual intercourse	255 (88.5%)
	Masturbation	09 (3.1%)
	Penile manipulation	18 (6.2%)
	Rolling in bed	01 (0.3%)
**Patients refused to provide data**		
	Signs and' symptoms	05 (1.7%)
	Hematoma	288 (100%)
	Detumescence	238 (82.6%)
	Snapping sound	220 (76.3%)
	Pain	191 (66.3%)
	Urethral bleeding	37 (12.8%)
	Acute urinary retention	01 (0.88%)
**Rupture of the tunica albuginea**		
	Unilateral	199 (69%)
	Bilateral	89 (31%)
**Urethral injury**		
	Partial	39 (13.5%)
	Complete	15 (5.2%)

**Figure 2:**
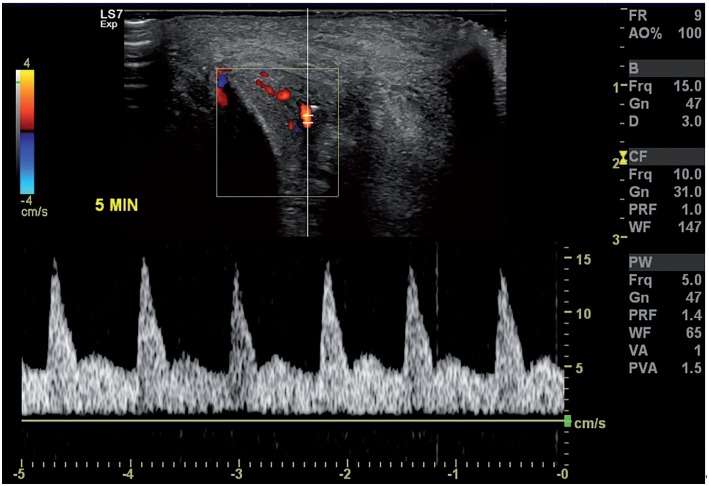
Penile color duplex Doppler ultrasound after pharmacological induced erection test through Alprostadil intracavernous injection excluding vascular disease in a patient with erectile dysfunction after a penile fracture.

**Figure 3:**
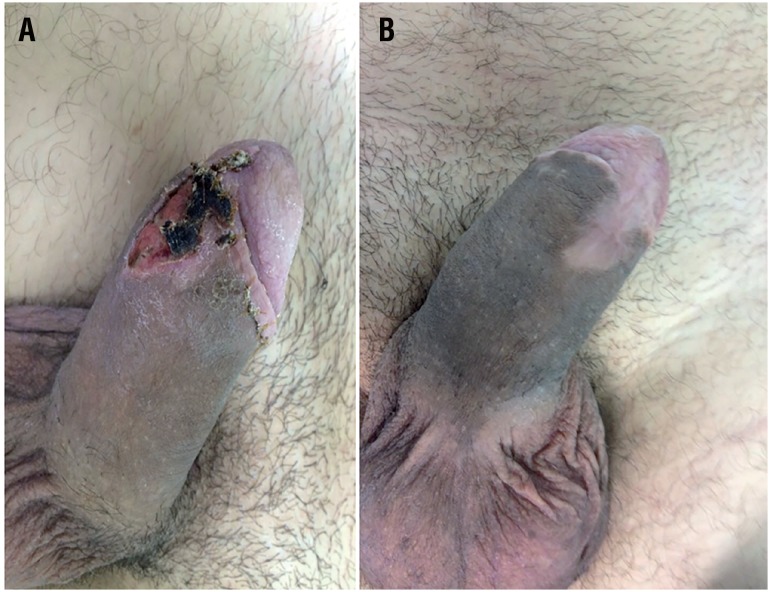
A-Necrosis of the surgical wound after circumcision B - Satisfactory evolution after conservative treatment with local dressings and with and secondary healing.

Of the 285 patients, 61 participated in follow-up of at least six months (mean 11.6). Forty-four (72.1%) patients developed penile nodule, 8 (13.1%) patients had penile curvature and 9 (14.7%) patients developed ED, of which 1 needed to perform penile color duplex doppler ultrasound with pharmacological induced erection test with alprostadil intracavernous injection to exclude vascular disease (Figure-2). Our data did not identify a statistical difference between the time of PF repair and ED or penile curvature rates. Of the 54 cases with associated urethral lesion, only two (3.7%) patients had complications (urethro-cutaneous fistula and subcutaneous abscess adjacent to the anastomosis area). Two (3.2%) patients presented necrosis of the operative wound (Figure-3). Postoperative complications are demonstrated in Table-2.

**Table 2:** Postoperative complications after penile fracture surgical treatment.

Complications	Cases (%)
Penile curvature	08 (13.1)
Erectile dysfunction	09 (14.7)
Penile nodule	44 (72.1)
Urethro-cutaneous fistula	01 (1.6)
Subcutaneous abscess	01 (1.6)
Necrosis of the operative wound	02 (3.2)

## DISCUSSION

While PF is an uncommon urological injury, its incidence is probably underestimated, since patients might not seek medical treatment in emergency rooms due to embarrassment. This fact, combined with the poor public health system in Brazil, may explain the long time lapse observed in this study between the occurrence of the trauma and hospital admission, which ranged from 2 to 504 hours (mean 18.5 hours). Even with treatment delay of 21 days, we did not identify a statistical difference between the time of PF repair and complications such as ED or penile curvature rates.

There are several causes of PF described in the literature in different regions of the world. The most common etiology in Western countries is sexual intercourse (4, 7). In Eastern countries, there is a higher incidence of cases associated with penile manipulation due to the practice of “thagaandan” in which the patients bends the distal portion of the penile shaft while holding the proximal part in place to achieve forced detumescence (2). Other practices, such as masturbation, falling on an erect penis, and rolling in bed have also been reported as causes in previous studies (8). El Atat et al. (9) described their experience with 300 cases of penile fractures and the etiology was masturbation in 180 cases (60%), rolling over in bed in 63 cases (21%), and sexual intercourse in 57 cases (19%). In our study, we observed that sexual activity was the most common mechanism of trauma, represented mainly by sexual intercourse (88.5%). As noted in a previous article by our group, the ‘doggy style’ and ‘man-on-top’ positions showed more associations with severe lesions such as bilateral fractures of the corpus cavernosum and urethral lesions (10).

PF is more common in younger individuals, with mean ages mostly in the fourth decade (7, 11). In our series, the patient's age ranged from 18 to 69 years (mean 38.2 years).

The recurrence of PF is even rarer, with few cases described in the international literature (12). We found only three (1%) patients with refracture. All presented the second episode with injury at the same point as the primary repair, but contralateral involvement was observed only in one case.

**Figure 4:**
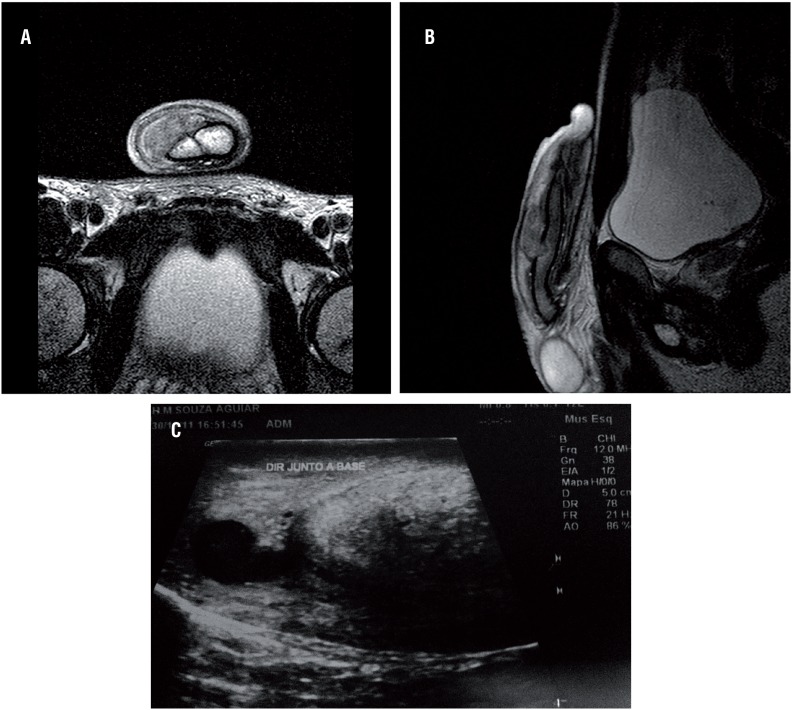
Patient with doubtful clinical picture of PF submitted to penile MRI demonstrating right corpus cavernosum base rupture with moderate hematoma in axial and sagittal images (A+B). Ultrasound demonstrating right corpus cavernosum base rupture with mild hematoma in another patient with doubtful clinical picture of PF (C).

For most authors, the diagnosis of PF is eminently clinical, with no need for additional tests since there is a typical clinical presentation. The typical triad of hematoma, detumescence, and snapping sound is a key diagnostic finding in the initial evaluation of these patients. According to Zargooshi (2), considering the excellent accuracy of clinical diagnosis, there is no need for any ancillary diagnostic test. Of 362 operated patients, 352 were intraoperatively proven to have PF and 10 had penile venous injury only. Diagnosis of PF in these 10 cases was made by our junior residents, who themselves operated on the patients. In a study conducted by Koifman et al. (4), the authors introduced the concept of penile trauma with low suspicion of PF in the assessment of doubtful cases. This new concept describes patients with a blunt trauma of the erect penis and no pain or immediate penile detumescence after the traumatic event, the presence of mild to moderate hematoma; and physical examination results, including palpation of the uninjured corpora cavernosa. A recent metanalysis reveals that 31 authors used no imaging, 22 authors used various image modalities to confirm the diagnosis: USG, cavernography, RGU and MRI (13). In our study, all patients showed penile hematoma upon admission, associated with detumescence in 82.6% of cases and a snapping sound in 76.3%. Only 6.6% doubtful cases underwent USG and 0.7% underwent MRI of the penis (Figure-4). RGU may show false-negative results in up to 28.5% of cases (14). Although RGU was performed in 25 of our cases, we believe that complementary examination is not necessary in cases of suspected urethral lesion in which penile degloving technique provides excellent exposure of the urethra and corpus cavernosum in all their extension. Urethral lesions are easily detected in the intraoperative period. Proof of this is that in the last 13 patients, RGU was performed in only one case.

Although according to most series the diagnosis of PF is made only by clinical findings, USG can be used to confirm the diagnosis and localize the site of the albuginea rupture and exclude the presence of urethral lesion.

This allows the access to the exact point of injury through a small skin incision avoiding the complications of degloving and postectomy (15) Mazaris (16), presented their experience with immediate surgical repair of eight patients with PF, using a midline ventral incision on the penile raphe. In six patients the diagnosis was confirmed by USG. According to the authors, this approach achieves good early and late results, has the advantage of direct access to both corpora cavernosa and the anterior urethra, with a minimal skin incision. More recently, Mao (17), described a study with 46 cases of PF treated using coronal proximal circular incision in 16 and local longitudinal incision in the other 30, according to the rupture location on USG. Fourteen of the 16 cases of circular degloving incision presented short-term postoperative foreskin edema but no postoperative complications were observed in any of the cases of local incision. The authors concluded that local longitudinal incision is sufficient to repair the tunica albuginea, without affecting the blood supply or lymph reflux, with low rate of complications. However, they defend the degloving when bilateral lesions of the corpora cavernosa and urethral injury are present. Circular sub-coronal incision and degloving of the penis with postectomy in uncircumcised patients was the technique standardized in our study. We found postoperative skin necrosis in two of 288 cases, accounting for only 0.6% of our total sample.

The presence of urethral injury associated with PF was reported as 3-38% (18). It is usually associated with high-energy trauma resulting in bilateral corpora cavernosa involvement. El-Ass-my et al. (19) reported 14 cases of urethral injury and all lesions were located at the same level as the corpus cavernosum, which were partial in 11 cases and complete in three. All patients had normal urinary flow except one, who developed relative urethral narrowing that required regular dilatation for one month. AAmong 312 cases of PF, Derouiche et al. (20) performed a retrospective study of a series of 10 cases of urethral lesion where no urethral stricture was noted after reconstruction.

In our study, urethral injuries were observed in 18.7% of cases, including 39 (13.5%) partial injuries and 15 (5.2%) total injuries. The complete rupture of the urethra was associated with bilateral injury in the corpus cavernosum in all cases. Only two (3.7%) patients had complications after urethral reconstruction (urethro-cutaneous fistula and subcutaneous abscess adjacent to the anastomosis area).

The surgical treatment of PF can lead to several long-term sexual complications. Zargooshi (2) evaluated 352 PF operated patients, and eight had sexual complaints at follow-up including premature ejaculation, ED, hypodesire disorder, anxiety, depression and marital conflict. El Atat et al. (9) described their experience with 300 cases of PF and observed complications in 40 patients (13.3%), of whom 14 (23.3%) developed penile curvature, 10 had penile nodules (3.34) and two suffered from erectile dysfunction (0.6%). In our study, of 61 patients that participated in follow-up of at least six months, nine (14.7%) developed ED and eight (13.1%) had penile curvature.

Some limitations of this study should be mentioned: The data are limited by the retrospective nature of the study but to our knowledge, this is the fourth largest case series published in the literature.

## CONCLUSIONS

PF has typical clinical presentation and does not need any additional tests in most cases. Hematoma and immediate penile detumescence after the traumatic event are the most common findings. Recurrent FP is extremely rare. Nevertheless, ipsilateral and even contralateral rupture of the prior PF may be present. Sexual activity is the most common cause. The ‘doggy style’ and ‘man-on-top’ positions are the most common and are generally associated with more severe lesions. Concomitant urethral injury should always be considered in cases of high-energy trauma, such as bilateral injuries in the corpora cavernosa and urethral bleeding or acute urinary retention. There is no ideal time of repair and a delay of a few days may be acceptable without interfering with the results. Surgical reconstruction produces satisfactory results. However, it can lead to complications, especially ED and penile curvature.
